# A Sustainable Approach for the Green Synthesis of Silver Nanoparticles from *Solibacillus isronensis* sp. and Their Application in Biofilm Inhibition

**DOI:** 10.3390/molecules25122783

**Published:** 2020-06-16

**Authors:** Priyanka Singh, Santosh Pandit, VRSS Mokkapati, Jørgen Garnæs, Ivan Mijakovic

**Affiliations:** 1 The Novo Nordisk Foundation, Center for Biosustainability, Technical University of Denmark, 2800 Lyngby, Denmark; prnksingh254@gmail.com; 2 Systems and Synthetic Biology Division, Department of Biology and Biological Engineering, Chalmers University of Technology, 41296 Gothenburg, Sweden; pandit@chalmers.se (S.P.); V.Mokkapati@evgroup.com (V.M.); 3 Danish Institute of Fundamental Metrology, Kogle Allé 5, DK 2970 Hoersholm, Denmark; jg@dfm.dk

**Keywords:** *Solibacillus isronensis*, silver nanoparticles (AgNPs), biofilm inhibition, *E. coli*, *P. aeruginosa*

## Abstract

The use of bacteria as nanofactories for the green synthesis of nanoparticles is considered a sustainable approach, owing to the stability, biocompatibility, high yields and facile synthesis of nanoparticles. The green synthesis provides the coating or capping of biomolecules on nanoparticles surface, which confer their biological activity. In this study, we report green synthesis of silver nanoparticles (AgNPs) by an environmental isolate; named as AgNPs1, which showed 100% 16S rRNA sequence similarity with *Solibacillus isronensis.* UV/visible analysis (UV/Vis), transmission electron microscopy (TEM), atomic force microscopy (AFM), dynamic light scattering (DLS), and Fourier-transform infrared spectroscopy (FTIR) were used to characterize the synthesized nanoparticles. The stable nature of nanoparticles was studied by thermogravimetric analysis (TGA) and inductively coupled plasma mass spectrometry (ICP-MS). Further, these nanoparticles were tested for biofilm inhibition against *Escherichia coli* and *Pseudomonas aeruginosa*. The AgNPs showed minimum inhibitory concentration (MIC) and minimum bactericidal concentration (MBC) values of 3.12 µg/mL and 6.25 µg/mL for *E. coli*, and 1.56 µg/mL and 3.12 µg/mL for *P. aeruginosa*, respectively.

Academic Editor: M. Concepción Gimeno

## 1. Introduction

Since ancient times, silver has been well known for its antimicrobial nature and, in various forms, such as silver nitrate, silver sulfadiazine, and metallic silver, has been applied for treatment of multiple infections [1]. This effect is the result of the generation of silver ions when the silver salts encounter water. Silver ions are powerful antimicrobials and are used for biomedical applications, such as biofilm inhibition on catheters, wound treatment, and so on. However, chlorides, phosphates, proteins, and other cellular components easily sequester them. In contrast, silver nanoparticles (AgNPs) are less susceptible to sequestration owing to their small size, and thus are more effective as antimicrobials agents. AgNPs have proven their efficacy against a variety of multidrug-resistant microorganisms [2,3,4]. The mechanism includes the adherence of AgNPs to the cell walls and membranes of microorganisms and may reach the cell interior. Following the damage of cellular structures and the production of reactive oxygen species (ROS), which in turn alter the mechanisms of signal transduction [5]. The most important properties of AgNPs, which play a key role here, are their exclusive size- and shape-tunable properties [6,7]. The size and shape of nanoparticles affect the bioavailability of silver ions in terms of dissolution or transport and interaction with biological targets [8]. Further, the available surface area and surface charge of nanoparticles also play a key role in interaction with biological moieties [9,10]. Owing to their antimicrobial nature, AgNPs are applied in designing a range of medical instruments, technologies, drug-carriers, and so on [2,3,4]. In addition, AgNPs became an attractive choice for the development of antimicrobial products, biosensor materials, fibers, and cosmetic and electronic products at industrial level [5].

There are various physiochemical methodologies available to prepare synthetic AgNPs. However, these methodologies possess some key limitations, such as absorption of toxic substances onto nanoparticles’ surface, or production of hazardous by-products. To overcome these limitations, “green” synthesis is employed, which involves the use of biological resources, such as microorganisms and plants products, for the nanoparticles synthesis. From the past few decades, bacteria came out as small nanofactories for producing these nanoparticles with remarkable properties, that is, biocompatibility and stability. The biocompatibility is a major advantage of green nanoparticles over synthetic nanoparticles, which is the result of the presence of an additional coating of biomolecules on the nanoparticles’ surface, often comes from source of synthesis. Thus, the green approach is considered as more sustainable and considered in the current study.

In medical terms, emergence of multidrug-resistant microorganisms and diminishing effectiveness of current antibiotics against them is a major problem [11]. Biofilm formation is one of the main mechanisms behind developing resistance to known antibiotics. Biofilms are complex association of microbes adhered to the biotic or abiotic surfaces encased within the matrix of their own synthesis called extracellular polymeric matrix (EPS) [12]. Biofilm formation initiates with the adhesion of free-floating bacterial cells to surfaces. There, they start producing EPS, collective matrix of polysaccharides, proteins, extracellular DNA (eDNA), and lipids to bridge over the bacterial cells and develop a complex 3D structure [13,14]. Bacterial cells in biofilms are demonstrated to have high tolerance to antimicrobial agents such as antibiotics and sanitizers compared with those in planktonic state. The insulating effect of the EPS matrix is considered to be the major reason behind the antimicrobial tolerance, as this layer limits the penetration of antimicrobial agents into the biofilm [14]. Hence, a high concentration of antimicrobial drugs is needed to destabilize biofilms and treat biofilm associated infections. A drastic increase in infections caused by multidrug resistant bacteria and the difficulties in their treatment have prompted the use of nanomaterials, especially AgNPs [15]. Many studies have confirmed strong bactericidal effects of AgNPs, suggesting their possible use for treatment of multidrug resistant bacterial infections [15]. AgNPs exert the antimicrobial effect through the release of free metal Ag+ ions in the aqueous solution, which bind proteins associated with the bacterial cell membrane and inhibit cell respiration and cell division, finally leading to cell death [4,16]. However, most of these findings are largely derived from the bactericidal effect of AgNPs against planktonic bacteria. Considering the impermeability of biofilms for classical antimicrobial agents, it is meaningful to evaluate the antibacterial activity of AgNPs against planktonic as well as bacterial cells in biofilms. Hence, the current study evaluated the antibacterial activity of AgNPs synthesized using an environmental isolate, AgNPs1, against both types of bacterial cells.

## 2. Materials and Methods

### 2.1. Materials

Analytical grade silver nitrate (AgNO_3_), tryptic soya agar (TSA), and tryptic soya broth (TSB) were purchased from Sigma-Aldrich Chemicals, St. Louis, MO, USA.

### 2.2. Isolation and 16S rRNA Gene Sequencing

Sampling of soil sample was done in sterile poly bags from Technical University of Denmark (DTU) field, Denmark. Next, to isolate the individual colonies, the soil sample was serially diluted in sterile 0.8% NaCl and then spread onto Typtic soy agar (TSA) plates [17,18]. The isolated colonies were screened on TSA plate containing 1 mM filter sterilized AgNO_3_, following incubation at 37 °C for 24 h. Molecular identification of the isolated strain was done using the 16S rRNA sequencing-based method [19,20]. The genomic DNA was extracted using a commercial genomic DNA extraction kit (Thermo Fisher Scientific, WA, USA). The 16S rRNA gene was amplified from the chromosomal DNA of the isolated strain using the universal bacterial primer set 27F, 518F, 800R, and 1512R [21]. The purified PCR products were sequenced by Eurofins, Galten, Denmark. Comparison of the 16S rRNA gene sequence of strain AgNPs1 with that of reference strains was carried out using the EzTaxon-e server, ChunLab, Seoul, South Korea [22,23].

### 2.3. Extracellular Synthesis of Silver Nanoparticles

The previously reported methodology was followed for extracellular synthesis of AgNPs [24]. Briefly, the isolate was inoculated into the 100 mL of sterile tryptic soy broth (TSB), following incubation at 37 °C, 120 rpm for 24 h [25]. After the incubation period, the bacterial cells were separated from the supernatant by centrifugation at 8000 rpm for 5 min. The 100 mL of cell free supernatant was further used for the synthesis of AgNPs.

For AgNPs synthesis, 1 mM filter-sterilized solution of AgNO_3_ was added into the 100 mL of culture cell free supernatant (reaction mixture). The reaction mixture further incubated for 1–2 days in an orbital shaker at 200 rpm and 37 °C. The synthesis was at first monitored visually for color change of the reaction mixture, following UV/Vis spectral analysis [26]. After the reduction, the nanoparticles were purified by centrifugation, first at 2000 rpm for 5 min, which allows removal of big particulates, followed by the centrifugation at 14,000 rpm for 15 min to collect the nanoparticles [24]. The obtained nanoparticles were washed thoroughly with water to remove the unconverted metal ions or any other constituents [27]. Finally, the nanoparticles were collected in the form of a pellet and used for analytical characterizations and in vitro biofilm inhibition applications [18].

### 2.4. Characterization of Silver Nanoparticles

#### 2.4.1. Nanoparticles Size and Shape Characterizations

UV/Vis spectrophotometer (UV/Vis) (6705 UV/Vis. Spectrophotometer, JENWAY) was used to confirm the transformation of silver metal ions into AgNPs by scanning the reaction mixture in the range of 300–700 nm. The optimization studies were conducted using UV/Vis [28]. The shape, size, and nature of nanoparticles were analyzed by transmission electron microscopy (TEM), by FEI Tecnai T20 G^2^ instrument operated at 200 kV. TEM further used for analyzing the selected area electron diffraction (SEAD) pattern of nanoparticles [29]. The sample preparation was done following liquid spotting on carbon coated copper grids, subsequently air drying before transferring it to the microscope [30]. Atomic force microscopy (AFM) (Park NX20 from www.parkafm.com) measurements were carried out in intermittent contact mode using standard probes of single crystal highly doped silicon with a radius of curvature of less than 30 nm (PointProbe Plus^TM^ or SuperSharpSilicon^TM^ Non-Contact AFM probes from Nanosensors). The standard uncertainty *u*(*d*) of the measured diameter is *u*(*d*) < 0.05 *d*.

Dynamic light scattering (DLS) (Zetasizer Nano ZS, Chuo-ku Kobe-shi, Japan) was used to study the nanoparticle size distribution and zeta potential. Hydrodynamic diameters and polydispersity index (PDI) were analyzed at 25 °C. As a reference, a dispersive medium of pure water with a refractive index of 1.330, viscosity of 0.8872, and dielectric constant of 78.5 was used [31]. The particles’ size and zeta potential of nanoparticles were conducted to measure the size distribution with a surface charge on the surface of nanoparticles.

#### 2.4.2. Nanoparticles Surface Study by Fourier Transform-Infrared Spectroscopy (FT-IR)

The FT-IR measurements were conducted as reported previously [28,32].

#### 2.4.3. Detection and Quantification of Nanoparticles by Single-Particle Inductively Coupled Plasma-Mass Spectrometry (sp-ICP-MS)

ICP-MS for size fractionation and quantification of nanoparticles was conducted as described in the literature [28,33].

#### 2.4.4. Nanoparticles’ Stability

Nanoparticles’ stability has been studied by various perspective, including a change in concentration of nanoparticles, thermal stability, pH, time stability in water, and stability in bacteriological media such as TSB and Luria broth (LB). For concentration stability, the nanoparticles’ concentration was measured for freshly prepared nanoparticles and after two week by sp-ICP-MS. Thermal stability of nanoparticles was determined by thermogravimetric analysis (TGA) analysis, for which the AgNPs in powder form were performed on a Discovery TGA, TA Instrument. For TGA analysis, nanoparticles were placed in an alumina pan and heated from 20 to 700 °C at a ramping time of 10 °C/min. The nanoparticles’ stability was further studied by scanning the samples in the 300–700 nm range, under the different ambient conditions. The nanoparticles were scanned before and after being kept for two weeks at room temperature, as well as in bacteriological media. Next, the absorbance was measured for the samples solution mixed with sodium hydroxide (NaOH) to achieve the range of pH from 4 to 10, to address the nanoparticles’ stability [34].

### 2.5. Silver Nanoparticles Application in Biofilm Inhibition

#### 2.5.1. Bacterial Strains and Culture Media

*Escherichia coli* UTI 89, *Pseudomonas aeruginosa* PAO1, *Staphylococcus epidermidis* ATCC 35984, and *Staphylococcus aureus* were used in this study to evaluate the antimicrobial efficacy of AgNPs. All the bacterial strains were tested to evaluate the effect of AgNPs on minimum inhibitory concentration (MIC), minimum bactericidal concentration (MBC), and biofilm formation. Precultivated *E. coli* and *P. aeruginosa* were treated with AgNPs to evaluate the antibiofilm activity. LB broth/agar was used as a growth medium for *E. coli* and *P. aeruginosa* and TSB broth/agar was used for *S. epidermidis* and *S. aureus.*

#### 2.5.2. Bacteriostatic and Bactericidal Activity

Bacteriostatic activity of AgNPs was evaluated by MIC and bactericidal effect was evaluated by MBC. MIC of AgNPs was realized by microdilution assay as described previously [35]. Briefly, overnight grown bacterial cultures were diluted to bacterial concentration of approximately 1–2 × 10^5^ colony forming unit (CFU)/mL. AgNPs concentrations ranging from 1.56 to 50 µg/mL in twofold dilution series were mixed with diluted bacterial suspension and incubated at 37 °C for 24 h and OD_550_ was measured. The MIC was defined by means of the lowest concentration of AgNPs, which inhibited the bacterial growth. For defining the MBC, 100 µL of the above described mixtures was streaked on agar plates and incubated at 37 °C overnight. MBC value was defined as the lowest concentration of nanoparticles, which prevented visible growth of bacteria on agar plates.

#### 2.5.3. Biofilm Inhibition Assay

The effect of AgNPs on biofilm formation was determined by biofilm inhibition assay, as described previously [28]. Briefly, overnight grown bacterial culture was diluted with respective medium to the final concentration of 1–2 × 10^6^ CFU/mL. The diluted bacterial suspension (200 µL) was added to a 96-well plate and incubated for 5 h at 37 °C. After 5 h of incubation, the old culture medium was replaced with fresh medium containing different concentrations of AgNPs and incubated further 19 h at 37 °C. After 24 h (in total) of biofilm growth, the medium was carefully removed and washed twice with sterile water without disrupting the biofilms to remove the free-floating bacteria. The biofilms were stained with 0.1% crystal violet for 20 min. The stain was removed and rinsed with sterile water for five times followed by drying at room temperature for 1 h. Absolute ethanol (200 µL) was added to the stained biofilms and the samples were agitated vigorously for 15 min to extract the stain. Optical density of extracted crystal violet was measured at 590 nm.

#### 2.5.4. Viability of Biofilm Bacteria with Silver Nanoparticle Exposure

Anti-biofilm activity of AgNPs was determined by counting the viable bacteria, scanning electron microscopic examination, and live/dead viability assay. The biofilms of *E. coli* and *P. aeruginosa* were formed on 15 mm cover glass as described previously. The overnight grown bacterial culture was diluted to get the inoculum containing bacterial concentration, 2–5 × 10^6^ CFU/mL. The diluted bacterial suspension was loaded on top of a cover glass and incubated at 37 °C for 24 h. After 24 h, old culture medium was replaced with fresh medium containing sterile water (negative control) or 1× MBC, 2× MBC, 4× MBC, or 8× MBC of AgNPs and further incubated for 24 h. After 24 h of nanoparticles exposure, biofilms were transferred to 0.89% of NaCl and homogenized using a probe sonicator for 20 s. The homogenized biofilm bacterial suspension was serially diluted and plated on LB agar plates and incubated overnight at 37 °C. The number of colonies was counted to determine the viability of bacterial cells (CFUs). To visualize live and dead cells, control and AgNPs treated biofilms were stained for 20 min with a mixture of 6.0 μM SYTO 9 and 30 μM KI from Live/Dead BacLight Viability kit L13152, (Invitrogen, Molecular Probes, Inc. Eugene, OR, USA). Fluorescence images were acquired using a Zeiss fluorescence microscope (Axio Imager.Z2m Carl Zeiss, Zena, Germany). Further, control and AgNPs treated biofilms were examined by scanning electron microscope (SEM). Biofilm samples were prepared for SEM as described previously [36]. Briefly, biofilms were fixed with 3% glutaraldehyde for 2 h and dehydrated with graded series of ethanol concentrations (40%, 50%, 60%, 70%, 80%, and 90% for 15 min each and with absolute ethanol for 20 min). The dehydrated biofilm samples were dried at room temperature overnight and coated with 5 nm of gold before SEM imaging, performed with a Supra 60 VP microscope (Carl Zeiss AG).

#### 2.5.5. Statistical Analysis

All experiments were performed with at least three biological replicates. Data are presented as the mean ± standard deviation (SD). The intergroup differences were estimated by one-way analysis of variance (ANOVA), followed by a post hoc multiple comparisons (Tukey test). Values were considered statistically significant at *p* < 0.05.

## 3. Results

### 3.1. Characterization of Strain

On the basis of molecular characterization, the strain AgNPs1 with 1449 bp sequence and NCBIaccession number MT522871 showed 100% identity with *Solibacillus isronensis*. *S. isronensis* has been reported as Gram-positive, rod shaped, spore forming bacterium isolated from upland soil [37].

### 3.2. Green Synthesis of Silver Nanoparticles

**Figure 1:**
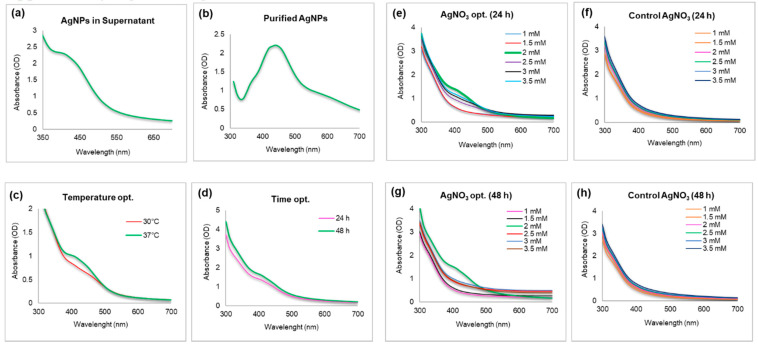
UV/vis spectra (**a**) unpurified AgNPs in supernatant and (**b**) purified AgNPs. Optimization studies based on UV/vis spectral analysis (**c**) temperature, (**d**) time, and (**e**–**h**) silver salt concentration.

The cell free supernatant showed transformation of silver ions to AgNPs gradually, which was visible owing to the change in color of reaction mixture from pale yellow to deep brown after the completion of the incubation period (48 h) (Supplementary Figure S1a) [38]. Next, the synthesis of nanoparticles was confirmed by scanning the nanoparticle suspension at a wavelength from 300–700 nm in a UV/Vis spectrophotometer (Figure 1a) [39,40]. The literature suggests that the spectra in the range of 350–500 nm corresponds to surface plasmon of AgNPs [3]. The synthesis was successfully confirmed, both directly in suspension, and with purified nanoparticles suspension (Figure 1b).

### 3.3. Optimization and Kinetics of Conversion of Silver Ions into Nanoparticles

**Figure 2:**
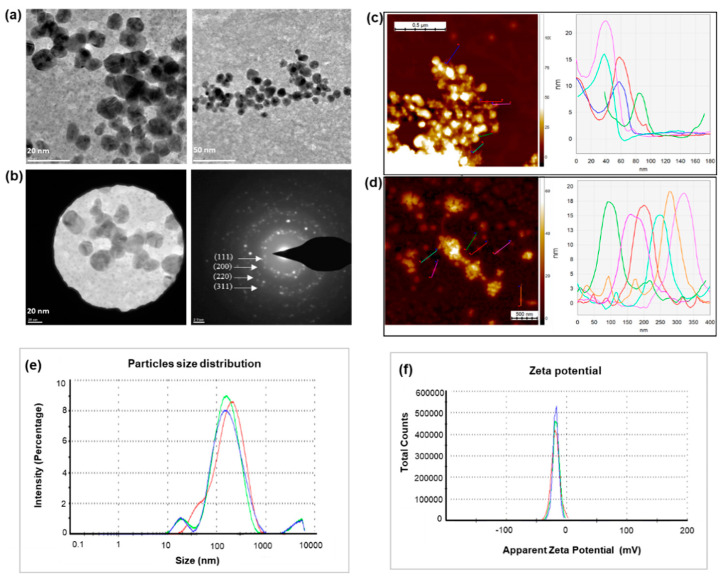
(**a**) Transmission electron microscopy (TEM) images of AgNPs showing the particles shapes, and (**b**) selected area diffraction pattern (SAED) of AgNPs with respective SAED apertures. The figures represent the size of nanoparticles of 80–120 nm with crystalline nature. AFM analysis of nanoparticle, (**c**) Z-height 128 nm, (**d**) Z-height 70 nm. (**e**) Particles size distribution with respect to the AgNPs intensity. (**f**) Zeta potential analysis, which shows the surface charge of nanoparticles with respect to total number of nanoparticles present in the solution. The study was done in triplicates and the results are an average of three analyses.

For temperature and time optimization, the results showed an optimal yield at 37 °C and 48 h (Figure 1c,d). For silver salt concentration optimization, the nanoparticles’ yield increased continuously until reaching a maximum of 2 mM salt, with a narrow peak, which indicates less polydispersity (Figure 2e,g). All the obtained spectra are in agreement with visual inspection of samples in cuvettes, as shown in Supplementary Figure S1d–g.

### 3.4. Characterization of Silver Nanoparticles

#### 3.4.1. Nanoparticles Size and Shape Characterization

TEM observations showed the quasi-spherical shape of nanoparticles with size range from 80 to 120 nm (Figure 2a). SAED was used to determine the nature of nanoparticles, which showed that particles were crystalline in nature [41]. The multiple electron diffraction patterns corresponding to the polycrystalline nature of the synthesized nanoparticles, which confirm to lattice planes of Bragg’s reflection (111), (200), (220), and (311) planes (Figure 2b) [17]. Next, AFM analysis was performed, which allows three-dimensional profiling, that is, measurement of nanoparticles height (Figure 2c,d). AFM analysis demonstrated that the nanoparticles are in the range of 70–130 nm.

Nanoparticles’ size distribution and surface charge were determined by DLS analysis. The hydrodynamic diameter observed was 127.8 nm with a polydispersity index (PDI) of 0.39 (Figure 2e). The relatively high PDI is indicative of a low monodispersity index. Nanoparticles exhibited negative zeta potential of −18.1 (Figure 2f), which confirmed the stable nature of synthesized nanoparticles.

#### 3.4.2. Nanoparticles Surface Study

**Figure 3:**
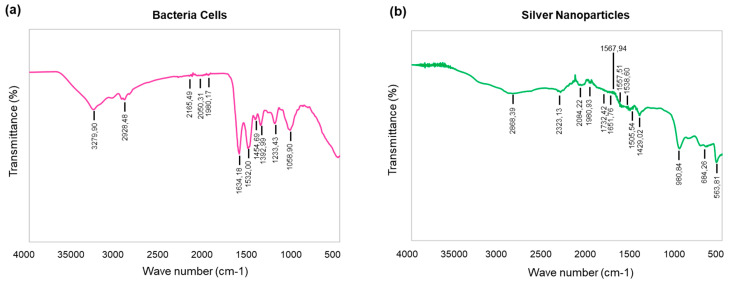
Fourier transform-infrared spectroscopy (FT-IR) spectra of bacteria cells and AgNPs for the identification of functional groups and interactions between said molecules and the nanoparticle surfaces. Note: FT-IR spectra of (**a**) bacteria cells and (**b**) AgNPs.

FT-IR measurements were conducted to analyze the presence of biological compounds on the nanoparticles capping layer [32]. The analysis of the FT-IR spectrum of bacteria cells and corresponding nanoparticles suggested extensive similarities between the cells and nanoparticles (Figure 3a,b) (Table 1).

#### 3.4.3. Nanoparticle Stability Study

**Figure 4:**
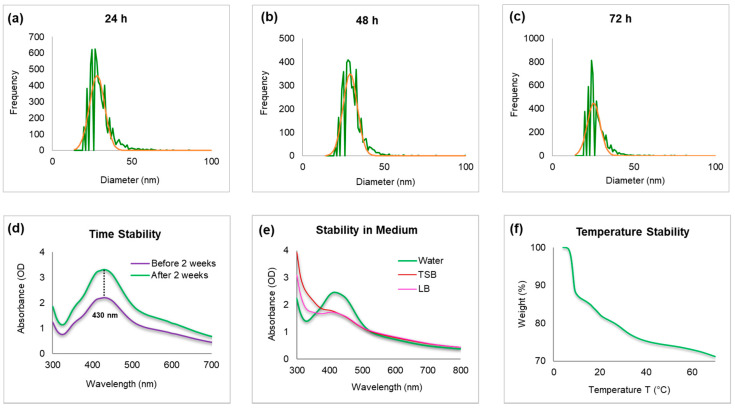
Inductively coupled plasma-mass spectrometry (ICP-MS) measurement of freshly prepared nanoparticles, which shows the size distribution histogram. (**a**) ICP-MS measurement of AgNPs after 24 h, (**b**) 48 h, and (**c**) 72 h of nanoparticles incubation to analyze the nanoparticles stability (the green color shows the frequency of size distribution and red is mean value). The dwell time was set to 50 µs and the scan time to 100 s. UV/Vis spectra for nanoparticles (**d**) time-dependent stability at a difference of two weeks, and (**e**) stability in bacteriological media. (**f**) Thermogravimetric analysis (TGA) measurement of nanoparticles, which shows the complete nanoparticles degradation at high temperature.

Nanoparticles’ concentration and size distribution were determined by sp-ICP-MS (Figure 4a–c). The measured total mass concentration was 1.14 ug/uL. The dissolved fraction was negligible (<0.1 ppb). The mean particle diameters was 46 nm. The measurements were repeated after one and two days and the results indicated no major deference in particles sizes, resulting in mean diameters of 46 nm, which indicated that the nanoparticles are stable. The particle number concentration stayed nearly the same and the storage time had no effect on AgNPs dissolution [28].

The nanoparticles’ stability was further confirmed by keeping the purified nanoparticles solution for up to two weeks at room temperature. The results indicated no significant shift in the UV/Vis spectrum of the nanoparticles solution, thus confirming the nanoparticles’ stability in the aqueous system (Figure 4d). The stability was further tested in bacteriological medium such as TSB and LB, and compared with aqueous medium stability (Figure 4e). The nanoparticles showed more stability in water as compared with growth medium. In addition, the effect of change in pH (range 4–10) was studied [24]. The nanoparticles solution was observed with and without NaOH, which results in no major change in the wavelength, thus confirming the stability of nanoparticles. To measure the thermal stability and weight loss profile of nanoparticles as a function of temperature, TGA measurement was conducted. Figure 4f demonstrates the weight loss profiles with increasing temperature. The complete degradation of nanoparticle between 200 and 400 °C indicates the actual organic phase materials adsorbed on the nanoparticles surface. A further increase in temperature up to 600 °C leads to nanoparticles’ degradation.

### 3.5. Biofilm Inhibition by Silver Nanoparticles

The antibacterial activity of synthesized AgNPs was initially tested against planktonic *E. coli*, *P. aeruginosa*, *S. aureus*, and *S. epidermidis* by means of MIC and MBC determination. The MIC and MBC values were observed to be 1.56 and 3.12 µg/mL against *P. aeruginosa* and 3.12 and 6.25 µg/mL against *E. coli*, respectively. Whereas *S. epidermidis* and *S. aureus* were observed to be less sensitive after AgNPs exposure, with MIC and MBC values of 25 and 50 µg/mL for *S. epidermidis* and 50 and 50 µg/mL for *S. aureus*, respectively (Table 2).

**Figure 5:**
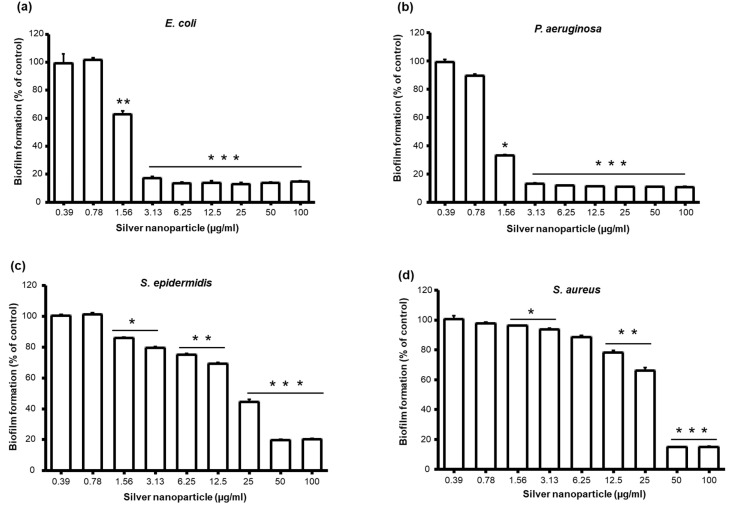
Effect of AgNPs on bacterial biofilm formation. (**a**) *E. coli*, (**b**) *P. aeruginosa*, (**c**) *S. epidermidis*, and (**d**) *S. aureus*. Data represent mean ± standard deviation error. * *p* < 0.05; ** *p* < 0.005; *** *p* < 0.0001.

The significant inhibition in biofilm formation by *P. aeruginosa* and *E. coli* was observed by 0.5× MIC concentration of AgNPs (Figure 5). On the other hand, biofilm inhibition by *S. aureus* and *S. epidermidis* was observed at much lower concentrations than of MIC values (which is 50 and 25 µg/mL, respectively) (Figure 5). Consistent with previous studies, higher sensitivity of gram-negative bacterial strains towards AgNPs was clearly observed compared with that of Gram-positive bacterial strains, which was attributed to the thickness of the cell membrane [42]. Gram-negative bacterial cells having thinner cell membrane compared with gram-positive bacterial cells are believed to be more prone to damage by AgNPs [43].

**Figure 6:**
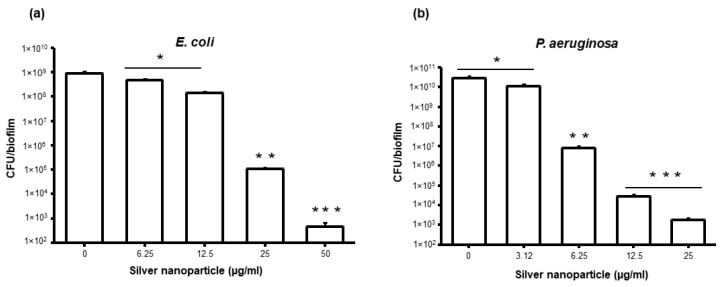
Colony forming unit (CFU) count of bacteria cells after treatment with AgNPs (**a**) *E. coli* and (**b**) *P. aeruginosa*. Data represent mean ± standard deviation error. * *p* < 0.05; ** *p* < 0.005; *** *p* < 0.0001.

Further, the efficiency of the synthesized AgNPs on biofilm formation by these bacteria was also evaluated. The inhibition in biofilm formation by MIC/sub MIC levels of AgNPs can be associated with gene expression levels that show significant bacteriostatic or partial bactericidal effect or inhibition. This might be the possible reason for the difference in the MIC/MBC with partial inhibition in biofilm formation by different bacterial strains. Further, along with strong bactericidal efficiency obtained from the MBC evaluation of AgNPs against *P. aeruginosa* and *E. coli*, antibiofilm activity was also evaluated. To test the antibiofilm activity, 24 h old biofilms of *P. aeruginosa* and *E. coli* were treated with MBC, 2× MBC, 4× MBC, and 8× MBC concentrations of silver nanoparticles. The viability was determined by colony counting method, scanning electron microscopy (SEM) analysis for any morphological changes, and visualization of live/dead bacterial cells in biofilm by fluorescence microscopy. The concentration-dependent loss in viability of bacterial cells was observed after 24 h of AgNPs treatment (Figure 6). 

**Figure 7:**
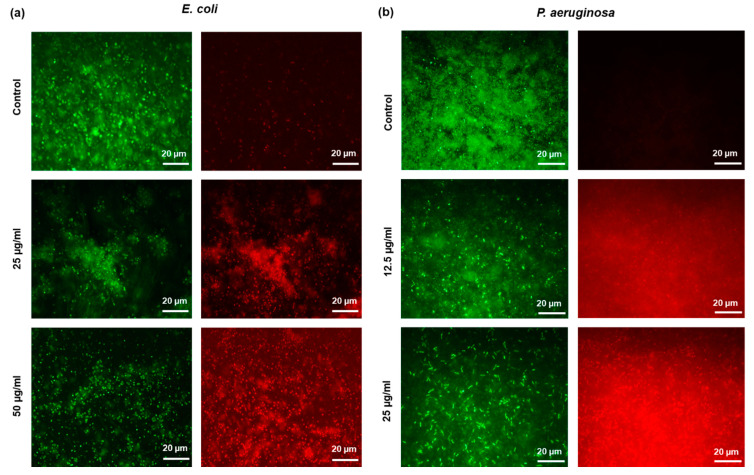
Live/dead staining image of biofilms observed by using fluorescence microscopy. (**a**) *E. coli* and (**b**) *P. aeruginosa*. Green shows live cells, red shows dead cells.

All the tested concentrations of AgNPs significantly reduce the viability of both bacterial strains and drastic loss in viability was observed on the biofilms that were treated with 4× MBC and 8× MBC. Even though significant reduction in viability of bacteria was observed, few viable cells were observed even in the biofilm treated with 8× MBC, suggesting that higher concentrations of AgNPs are needed for complete biofilm eradication. To confirm the ratio of live/dead cells in biofilms treated with AgNPs, control and nanoparticle treated biofilms were stained with live/dead stain and examined by fluorescence microscope [28]. Images acquired from fluorescence microscopy shows drastic decrease in live cells and significant amount of cell death in nanoparticle treated biofilms, suggesting the severe toxicity of nanoparticles towards both *P. aeruginosa* and *E. coli* biofilm cells (Figure 7).

**Figure 8:**
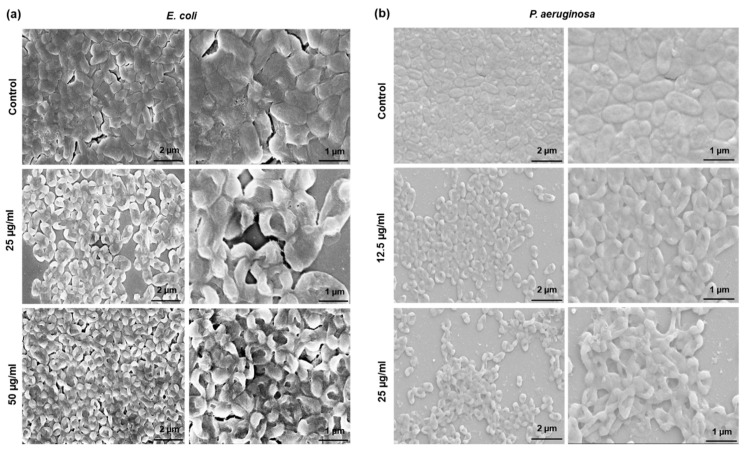
Scanning electron microscopy (SEM) image of bacteria cells treated with different concentrations of AgNPs. (**a**) *E. coli,* and (**b**) *P. aeruginosa*.

The morphological disruption is mainly owing to the disintegration of cell membrane for most of the bacterial cells that are treated with AgNPs (Figure 8). The disintegration of bacterial cell membrane by AgNPs might be owing to the interaction of Ag^+2^ ions with negatively charged microbial cells, which in turn generate multiple pores on the cell membrane, leading to the leakage of intracellular contents, which ultimately results in permanent cell death [44].

## 4. Discussion

Generally, green synthesis employs plants and microorganism products to act as reducing agents, and have always been reported as less complicated and producing highly effective nanoparticles. In contrast, electrochemical methodologies require a lot of efforts and sources, such as electrodes, current, reducing chemicals, toxic or hazardous solvents, and so on [45]. In the current study, we explored green methodology of AgNPs synthesis using a bacterial strain isolated from soil sample.

Nanoparticles’ production from micro nanofactories have been reported in both intracellular and extracellular ways. Generally, the microorganisms showing resistance to metal ions tend to produce those metal nanoparticles effectively. In the current study, the isolated strain showed growth in the presence of silver salt, which suggested that the isolate was resistant to silver ions. On the basis of 16S rRNA sequence similarity, the isolate was named *Solibacillus isronensis* sp. strain AgNPs1. For nanoparticles’ synthesis, following 24 h growth, the cells were separated from the supernatant by centrifugation and supernatant was further used to for extracellular nanoparticles’ synthesis. The isolated started showing the reduction first at 24 h; however, the reaction time was stretched up to 48 h to ensure the complete reduction of silver salt. After 48 h of incubation, the color of supernatant was transformed from pale yellow to deep brown, which is owing to the surface plasmon resonance by AgNPs produced in the medium (Supplementary Figure S1a) [38]. Singh et al. recently showed the synthesis of 10–40 nm AgNPs from *Pseudomonas* sp. [18]. The cause of reduction could be the biological components produced in the supernatant, such as polysaccharides, proteins, enzymes, amino acids, and so on. However, the exact synthesis mechanism is still unclear [46]. Most importantly, the isolated showed AgNPs production extracellularly, which makes the process of particles purification less tedious as compared with intracellular synthesis. This is because intracellular synthesis requires additional steps of membrane lysis and particles isolation from cellular matrix. To our knowledge, this is the first report of using the genus *Solibacillus* for efficient extracellular synthesis of AgNPs.

Reaction parameters, such as temperature, time, and concentration of silver salt, have been taken into consideration to evaluate the possibilities for complete and efficient reduction. As shown in the results, the maximum reduction of silver salt to nanoparticles was observed at 37 °C, 48 h, and 2 mM silver salt conc. Any increase in temperature, time and salt concentration leads to a decrease in bacterial growth and a major shift in peaks of AgNPs spectrum. The color difference of reaction medium corresponding to the spectrum is clearly visible in Supplementary Figure S1d–g [28]. The TEM and AFM sizes of nanoparticles were also in alignment. The particle size measurement showed 127.8 nm with 0.39 PDI, which is different from the TEM and AFM sizes (Figure 2). This is owing to the fact that DLS measures the hydrodynamic particles size in aqueous system, whereas TEM measures the core size of nanoparticles. In FT-IR (Figure 3), the spectra of bacteria cells are characterized by O-H band at 3279.99. The C-H stretching was observed between 2928.48 and 2868.39 cm^-1^ for cells and silver nanoparticles. The alkyne group was observed at 2165.18, 2050.31, and 1098.17 for cells and at 2323.13, 2084.22, and 1980.93 for silver nanoparticles [47]. Moreover, C=C double-bond stretching was shown in both samples in the range from 1634.18 to 1505.54. CH_3_, CH_2_ asymmetric deformation was observed at 1454.69 and 1392.99 for cells and 1429.02 for nanoparticles. C-O stretching at 1058.90 and 980.84 cm^−1^ was detected in both cells and nanoparticles, respectively [28]. C-C deformation was observed in nanoparticles samples at 684.26 and 563.81 cm^−1^. Water-soluble biomolecules and active enzymes present in the supernatant play major roles in reduction and forming capping agents around nanoparticles surface, which play a major role in the stabilization of synthesized nanoparticle. In green synthesis, the capping layer naturally from around the nanoparticles surface by the help of various biomolecules comes from biological resources. These capping layers not only provide the stability, but also make the nanoparticles biocompatible in nature as compared with chemically or physically synthesized nanoparticles. The nanoparticles’ stability results also showed constant alignments in peaks at different conditions, which suggest that the particles are stable and avoid agglomeration [48,49].

Bacterial cells embedded in biofilms are more resistant to antibiotics. The resistant is because of the inactivation of antimicrobial agents by EPS compounds, reduced penetration of antibiotics in biofilm matrix, and altered metabolic rate of bacterial cells within biofilms. AgNPs have shown their wide range of antimicrobial activity against many biofilm forming microorganisms [46]. AgNPs interact with biofilms first by transporting the nanoparticles to the biofilm–fluid interface, attachment to the biofilm surface, and ultimately migration within the biofilm. Size plays a critical role in the interaction of nanoparticles with biofilm,;in general, the relative self-diffusion coefficients decrease exponentially with the nanoparticles’ size [50]. In the current study, the green nanoparticles showed biofilm inhibition and lethal action against *E. coli* and *P. aeruginosa.* The SEM observation of nanoparticles treated cells showed complete disruption of cell morphology with regard to its shape, wall, and size; Figure 8. The possible action mechanism of nanoparticles could be damage to cell wall and cell membrane, intracellular penetration, and oxidative stress [46]. Loo et al. showed the 90% reduction of biofilms produced by *P. aeruginosa* after exposure to 600 μg/mL AgNPs [51]. However, in the current study, 25 μg/mL of AgNPs showed *P. aeruginosa* biofilm reduction; this could be attributed to the high purity and effectiveness of green AgNPs. In addition, 50 μg/mL of AgNPs showed biofilm inhibition against *E. coli.* Research suggests that nanoparticles’ surface charge and functional groups play a critical role in interaction with EPS components and in the efficacy of nanoparticles. On the interaction of nanoparticles with biofilm environment, which has active organic molecules, a corona complex forms around nanoparticles surface and the nature of this corona influences the nanoparticles–biofilm interactions [52]. In this study, the effectiveness of AgNPs is probably owing to the nanoparticles accumulation in the biofilm, and to their inability to agglomerate inside cellular atmosphere because of their stable nature [52]. The stable nature of nanoparticles is due to the formation of the capping layer around the nanoparticles’ surface, which often comes from the biological moieties present in the reaction mixture. Thus, the particles ultimately show high efficacy to conquer the negative consequences of biofilms [53].

## 5. Conclusions

This work demonstrated a sustainable approach for the extracellular green synthesis of AgNPs from an environmental isolate AgNPs1, which showed 100% identity with *S. isronensis* based on 16S rRNA sequencing. The synthesized AgNPs were crystalline, stable, and showed biofilm inhibition in *E. coli* and *P. aeruginosa*. Thus, the AgNPs synthesized using *S. isronensis* AgNPs1 enable the control of biofilms. These findings open perspectives for future investigations and the possibility of applications of green nanoparticles as biofilm inhibitors in the areas of healthcare and medicine.

## Figures and Tables

**Table 1:** Fourier transform-infrared spectroscopy (FT-IR) spectra of bacteria cells and silver nanoparticles (AgNPs).

Type of Bond	Cells Wavenumber (cm^−1^)	AgNPs Wavenumber (cm^−1^)
O-H strech	3279.90	-
C-H strech	2928.48	2868.39
Alkyne group	2165.49, 2050.31, 1098.17	2323.13, 2084.22, 1980.93
C=C strech, amide C=O strech	1634.18, 1532.00	1732.42,1651.76,1567.94–1505.54
CH_3_, CH_2_ asymmetric deformation	1454.69, 1392.99	1429.02
C-O strech	1058.90	980.84
C-C deformation	-	684.26, 563.81

**Table 2:** Minimum inhibitory concentration (MIC) and minimum bactericidal concentration (MBC) of silver nanoparticles against *E. coli*, *P. aeruginosa*, *S. epidermis,* and *S. aureus*.

Strains	MIC (µg/mL)	MBC (µg/mL)
*E. coli*	3.12	6.25
*P. aeruginosa*	1.56	3.12
*S. epidermidis*	25	50
*S. aureus*	50	50

## Supplementary Materials

The following are available online at, Figure S1: Visible picture of AgNPs production in (a) cell free supernatant and control (only media), after the incubation period. Visible observation of optimization parameters for AgNPs production: (b) temperature optimization, (c) time optimization, (d) salt (AgNO_3_) optimization after 24 h, (e) control after 24 h, (f) salt (AgNO_3_) optimization after 48 h, and (g) control after 48 h.
