# Supplementary figures and images for: A Sustainable Approach for the Green Synthesis of Silver Nanoparticles from Solibacillus isronensis sp. and Their Application in Biofilm Inhibition
Source: Molecules. 2020 Jun 16;25(12):2783. doi: 10.3390/molecules25122783 (PMC7355478; doi:10.3390/molecules25122783)

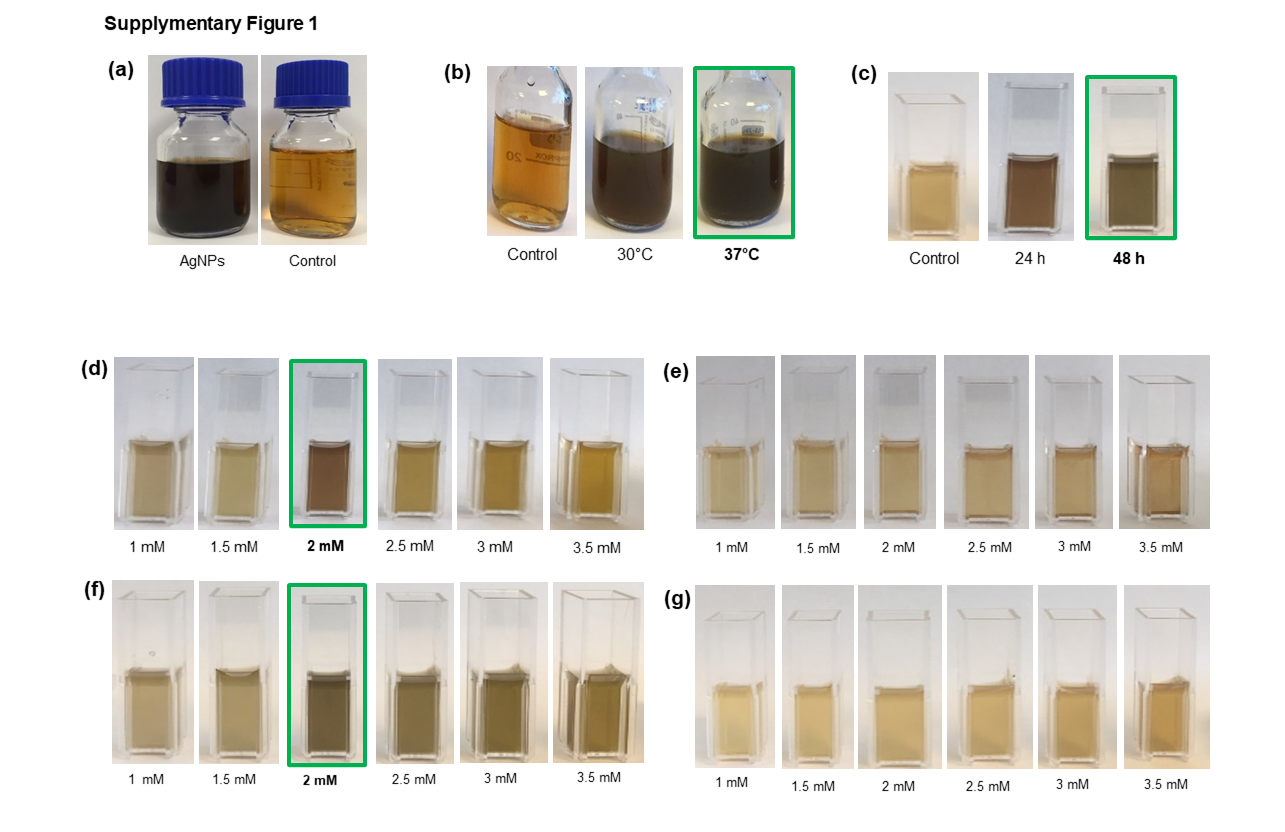

Supplement: Supplementary file 1 [file molecules-25-02783-s001.zip › molecules-798022-supplementary.tif]
